# Cecal Volvulus in a child with congenital dilated cardiomyopathy: A case report

**DOI:** 10.1016/j.ijscr.2019.10.008

**Published:** 2019-11-23

**Authors:** Ahmed E. Shehata, Mohamed A. Helal, EzzElDien A. Ibrahim, Basma Magdy, Mohamed El Seoudy, Muayad Shaban, Heba Taher

**Affiliations:** Pediatric Surgery Department, Cairo University, Egypt

## Abstract

**Introduction:**

Cecal volvulus is an extremely rare cause of intestinal obstruction in the pediatric age group and its incidence is unknown.

**Presentation of case:**

We present a unique case of cecal volvulus in a 3 year old female with congenital dilated cardiomyopathy.

**Discussion:**

Cecal mobility due to malfixation and malrotation is the main cause. Cecal Volvulus usually presents with constipation, abdominal pain and distention. Common complications entail strangulation, ischemia and gangrene. Cecal volvulus has been reported in association with various abnormalities. However, Incidence of cecal volvulus in a child with congenital dilated cardiomyopathy has not been reported. Surgery is the mainstay of management.

**Conclusion:**

Cecal volvulus should be kept in mind in a child presenting with signs suggestive of intestinal obstruction.

## Highlights

• Cecal volvulus is a very rare cause of intestinal obstruction in children.
• Novel association between Congenital dilated cardiomyopathy and cecal volvulus.
• Cecal volvulus should be suspected in a child presenting with bilious vomiting.

## 1 Introduction

Cecal volvulus (CV) is an extremely rare cause of intestinal obstruction in the pediatric age group and its incidence is unknown [1]. Cecal mobility, due to malfixation and malrotation, is the main cause [2]. CV usually presents with constipation, abdominal pain and distention [1,3]. Common complications entail strangulation, ischemia and gangrene [4]. Here we present a unique case of cecal volvulus in a 3 year old female with congenital cardiomyopathy. The work has been reported in line with the SCARE criteria [5].

## 2 Presentation of case

**Fig. 1:**
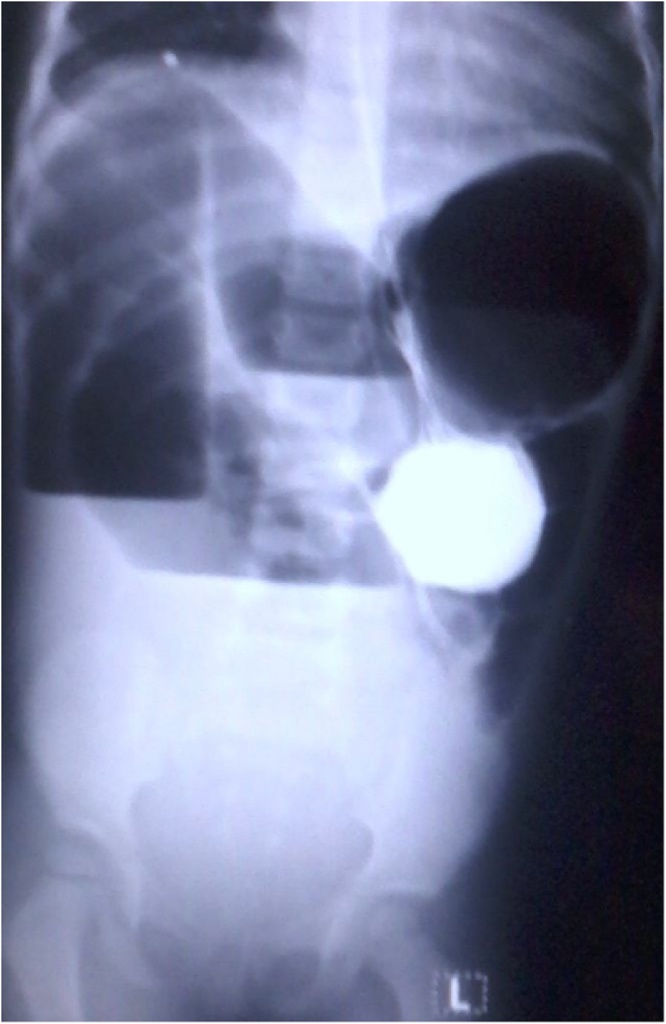
Abdominal X-ray with contrast showing multiple air fluid levels.

**Fig. 2:**
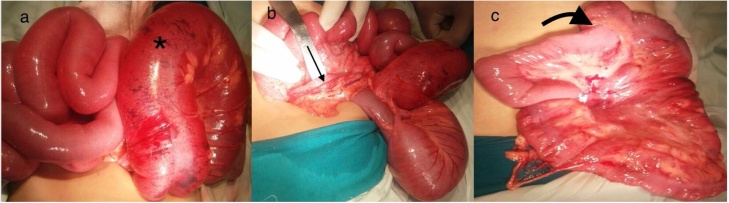
Intraoperative pictures showing the Cecal volulus (* in a) and the band extending between the base of the cecum and and the hepatic flexure (arrow in b) and after release of the band, appendectomy and De-torsion (thick arrow in c).

A 3-year-old female, with a history of mild congestive dilated cardiomyopathy, presented to our ER with constipation of 3 days duration, and a history of repeated attacks of bilious vomiting and abdominal bloating. There was no history of colics and her bowel habits were normal before this event. Physical examination revealed dehydration and her abdomen was distended, lax and moved freely with respiration. There was neither tenderness nor abdominal guarding. Her abdomen was resonant on percussion with an empty rectum. Labs revealed a total leukocytic count of 3.6 × 10^9^/L, Hemoglobin was 11.1 gm/dl and the platelet count was 343 × 10^9^/L. Abdominal X-ray with contrast was performed (Fig. 1). Exploratory laparotomy was decided and it revealed cecal volvulus, with a band extending from the base of cecum to the hepatic flexure, with collapsed proximal and distal loops (Fig. 2). Release of the band with detorsion, appendectomy and cecopexy were performed (Fig. 2). Postoperatively the patient stayed in the ICU for 7 days and ileus persisted for 4 days. She started oral intake on the 5th day and was discharged on the 7th day with no complications. The patient was doing well during her 2 year follow up period.

Of note, the patient was born at term with no complications at birth. She had a hemangioma on the lower left eyelid that failed laser treatment and resolved with propranolol.

Her dilated cardiomyopathy was discovered on rounding check up and is medically controlled using furosemide, captopril, spironolactone, digoxin and aspirin.

Her echocardiogram showed situs solitude, normal venous drainage, atrioventricular concordance, ventriculoarterial concordance and intact interatrial and interventricular septum. The Left ventricle was hugely dilated with global impairment of systolic function, ejection fraction was around 40%, with no septal hypokinesia and there was an associated grade II-III secondary mitral regurgitation. Additionally, the cardiac valves, aortic arch, coronary arterial origin and size were normal. There was no detected coarctation of the aorta (CoA), patent ductus arteriosus (PDA) nor pericardial effusion.

There is paternal consanguinity and the parents gave a history that the patient’s older sister underwent an operation at the age of 18 months for intestinal obstruction; however the patient records and details of the operation were not available.

## 3 Discussion

Cecal volvulus occurs due to an abnormally mobile cecum due to improper developmental fixation or malrotation of the cecal mesentery [2,3]. CV is an uncommon cause of intestinal obstruction in adults constituting about 1% of the cases and it accounts for about 30% of cases of colonic volvulus [2,6]. However, it’s extremely rare in children and its true incidence in not known.

The clinical presentations of patients with CV vary depending on the extent of involvement and duration of the twist, with chronic constipation, generalized abdominal bloating and vomiting being the usual symptoms. CV can lead to serious complications including strangulation, gangrene, perforation and peritonitis [3,4,6]. Due to the nonspecific nature of the presenting symptoms, early detection is hard and radiography is often needed [1,3]. In the case presented here, abdominal X-ray revealed multiple air-fluid levels which prompted the decision for exploratory laparotomy. Other radiologic modalities for detecting CV include contrast enema, Computed tomography (CT) and ultrasound. Surgical management is the mainstay of treatment of CV. Although decompression by colonoscopy has been reported as a line of management, it has a high failure rate [3]. Various modalities include manual detorsion, cecopexy, cecostomy and colectomy by open or laparoscopic approaches [6,7]. In the case presented, manual detorsion and cecopexy were performed along with appendectomy.

CV has been reported as a postoperative complication of right nephrectomy and in association with Cornelia de Lange syndrome [1,[8], [9], [10]]. Cornelia de Lange syndrome is characterized by intrauterine and postnatal growth retardation, mental retardation, characteristic craniofacial features and limb anomalies [8]. Congenital heart disease and structural heart defects have been reported in patients with this syndrome, in addition to minor defects including patent ductus arteriosus and patent foramen ovale, with a prevalence ranging from 14% to 70%. However, congenital dilated cardiomyopathy was not reported as an association [11]. As the patient didn’t present any of the characteristics of the syndrome, the presence of congenital dilated cardiomyopathy in this patient constitutes a novel association between and CV and congenital cardiomyopathy.

## 4 Conclusion

Although CV is a rare cause of intestinal obstruction in pediatrics, it should be kept in mind in a child presenting with chronic constipation and abdominal bloating. Early diagnosis is crucial to prevent the potentially fatal complications of CV. The association of congenital dilated cardiomyopathy and CV should also be kept in mind.

## Sources of funding

This research did not receive any specific grant from funding agencies in the public, commercial, or not-for-profit sectors.

## Ethical approval

Our study is exempted from ethical approval in Institutional Review Board of our hospital.

## Consent

We have written informed parental consent to publish this report.

## Author contribution

Ahmed Shehata: Literature Review and drafting manuscript.

EzzElDien Ibrahim: literature review and drafting manuscript.

Mohamed Helal: literature review editing and revising the manuscript.

Basma Magdy: operating on the patient and presenting the case.

Mohamed Elseoudy: operating on the case.

Muayad Shaban: literature search discussion data collection.

Heba Taher: Clincal supervision, drafting and revising the manuscript.

## Registration of research studies

This is a case report and not a human study. It is exempt from registering.

## Guarantor

Heba Taher.

## Provenance and peer review

Not commissioned, externally peer-reviewed.

## Declaration of Competing Interest

We declare no conflict of interest.
